# Angiographic characteristics of coronary artery disease in patients undergoing diagnostic coronary angiography at a tertiary hospital in Tanzania

**DOI:** 10.1186/s12872-024-03773-7

**Published:** 2024-02-26

**Authors:** John R. Meda, Happiness L. Kusima, Ng’weina F. Magitta

**Affiliations:** 1 Department of Internal Medicine, School of Medicine & Dentistry, University of Dodoma, Dodoma, Tanzania; 2 Department of Cardiology, Benjamin Mkapa Hospital, Dodoma, Tanzania; 3 Department of Biochemistry & Clinical Pharmacology, Mbeya College of Health & Allied Sciences, University of Dar es Salaam, Mbeya, Tanzania

**Keywords:** Coronary artery disease, Chest pain, Acute coronary syndrome, Coronary angiography, Risk factors

## Abstract

**Background:**

Coronary artery disease (CAD) is an important cause of global burden of disease. There is a paucity of data on the burden and risk factors for CAD in sub-Saharan Africa (SSA), despite the rising trends in the shared risk factors across regions. The recent introduction of cardiac catheterization laboratory services in SSA could shed light on the burden of CAD in the region. We aimed to assess the angiographic characteristics among patients undergoing diagnostic coronary angiography (CAG) at a single tertiary care hospital in Tanzania.

**Methods:**

This study was a retrospective chart review. A total of 728 patients  ≥ 18 years of age who underwent CAG from January 2020 to December 2022 were recruited into the study. Basic demographic variables, risk factors and clinical characteristics including CAG findings were obtained from the registry. In addition, CAG images were retrieved for assessment of angiographic features. The luminal vessel stenosis was assessed based on eyeballing and the degree of obstruction was agreed by two independent and experienced cardiologists. The coronary stenosis of ≥ 50% was considered significant for obstructive CAD. The study was approved by the local ethics committee.

**Results:**

Of patients who were recruited into the study, 384 (52.23%) were female. The study participants had a mean age of 59.46 ± 10.83 standard deviation (SD) and mean body mass index (BMI) of 31.18 kg/m^2^. The prevalence of CAD of any degree was estimated at 24.43% (34.18% in male, 15.50% in female), while that of obstructive CAD was 18.27%. Forty six percent of those with obstructive CAD had multiple vessel disease (MVD). Nearly 77% of patients were found to have ≥ 50–70% luminal stenosis and while those with ≥ 70% luminal coronary artery stenosis constituted 56.65%. Right coronary artery (RCA) was the most commonly affected vessel, accounting for 36.84% when any vessel disease or 56% when single vessel disease were considered. Being 65 years or older and comorbidity with type 2 diabetes (T2D) were independent risk factors for developing CAD.

**Conclusion:**

There is a high prevalence of obstructive CAD among patients undergoing diagnostic CAG in Tanzania, with male gender preponderance and increasingly higher in older age, often with severe disease. A large, prospective study is needed to provide epidemiological and clinical data for developing a locally-relevant cardio-preventive strategy for CAD intervention in Tanzania.

## Background

The 2021 report on Global Burden of Disease (GBD) study indicate Coronary artery disease (CAD) as the leading cause of global burden of disease, accounting for 9.44 million deaths and 185 million disability-adjusted life years (DALYs) [1]. There are few reports on the burden and risk factors of developing CAD in Sub-Saharan Africa (SSA). However, the current global reports and regional estimates indicate the rising trends in the shared risk factors for chronic non-communicable diseases (NCDs), and therefore likely to substantially contribute to the silent epidemic of CAD in SSA [2, 3]. Regrettably, lack of awareness, underreporting and misdiagnosis of CAD could account for observed fallacy of its low burden in SSA [4]. The global prevalence of CAD is estimated at 1.74% as reported by Khan MA et al. [5]. This is comparable to the available few population-based studies conducted in SSA. For instance, studies conducted in South Africa and Nigeria reports the population prevalence of CAD to be 1.29% and 1.6% respectively [6, 7] .

The hallmark of CAD is atherosclerosis resulting from vascular endothelial injury which elicits and promotes chronic inflammatory response, increased oxidative stress, fibroblasts proliferation, fibrin/collagen deposition and vascular smooth muscle remodeling [8–10]. The well characterized risk factors for CAD include hypertension, type 2 diabetes (T2D), LDL hypercholesterolemia, obesity and sedentary lifestyle, smoking/tobacco use, alcohol abuse and consumption of high energy, low fibre, processed foods [8, 10].

A recent review by Shehu, MN et al. reports a steady increase in CAD prevalence and rising risk factors [11]. These traditional risk factors works continuously in an orchestrated manner over many years to promote the pathogenesis of atherosclerotic cardiovascular diseases (ASCVD) [9]. However, it’s argued that the *nidus* for future development of ASCVD could begin in early childhood and potentially *in utero* where fetal programming and pre-conditioning are believed to occur to influence ASCVD susceptibility [12]. Thus, this novel insight prompts a comprehensive life course approach in the control of ASCVD.

Coronary artery reperfusion therapy remains the gold standard for the management of acute coronary syndrome (ACS) together with a selected group of patients with chronic coronary syndrome (CCS) [13–15]. However, this intervention requires specialized laboratories, material resources and expertise which are limited in the majority of tertiary care facilities in SSA [16]. Coronary angiography (CAG) serves to determine the type and number of vessels involved along with the extent of stenosis; critical parameters for guiding coronary intervention modality. The recent introduction of CAG services in a few centres in SSA provides an important tool for gaining insight on the extent of CAD, particularly the angiographic characteristics necessary for guiding tailored intervention and overall improvement of patients’ care [16]. Arguably, the increasing advancement and investment in cardiovascular medicine is likely to pave the way to better diagnosis and intervention while impacting on clinical research and adoption of the “best buys” in clinical care of patients with CAD.

This study was conducted to assess the angiographic characteristics among patients undergoing diagnostic CAG at a single tertiary care hospital located in Dodoma; a rapidly urbanizing and a newly enforced an administrative capital city of the United Republic of Tanzania.

## Methods

This study was a retrospective chart review which was based on the existing “Cath Lab” registry at Benjamin Mkapa Hospital in Dodoma, Tanzania. The study patients were selected from the “Cath Lab” registry, which included all patients who underwent diagnostic CAG for CAD. A total of 728 eligible patients aged 18 years and above, with near complete records, who underwent diagnostic CAG over a three year period, from January 2020 to December 2022 were recruited into the study. Basic demographic variables, risk factors and clinical characteristics including CAG findings were obtained from the registry. CAG findings were categorized based on American College of Cardiology/American Heart Association (ACC/AHA) and European Society of Cardiology (ESC) guidelines [13, 14]. The CAG images were retrieved and one cardiologist who did not participate in CAG procedures was responsible for the assessment of angiographic characteristics on the retrieved images and the results were compared with those recorded in the registry. A consensus was sought through a discussion, with the third independent cardiologist, in cases where there was a discrepancy between the reported and the recorded angiographic features. The assessment of the coronary artery stenosis was performed based on the eyeballing method and the degree of obstruction was agreed by two independent experienced cardiologists. The obstructive CAD was defined as the luminal stenosis of ≥ 50% in the coronary artery.

The study was approved by the University of Dodoma, Institutional Research Review Committee, with reference number MA.84/261/65/27, and was conducted in accordance to the principles of the revised Declaration of Helsinki.

### Statistical analysis

Data were analyzed using STATA version 18 (StataCorp., TX, USA). Categorical variables were summarized using counts and percentages while continuous variables were summarized using means and standard deviations (SD). Cross-tabulation with χ^2^ test were used to describe the association between age, sex and other risk factors with specific angiographic characteristics as outcome variables. Bivariate and multivariable logistic regression analyses were performed to identify factors associated with specific angiographic characteristics. All variables with a *p*-value < 0.2 in the univariate model were included in the multivariable models to estimate the adjusted odds ratio. A *p*-value less than 0.05 was considered statistically significant.

## Results

### Selection of patients from the hospital registry

### Patients’ demographic variables and clinical characteristics

**Fig. 1:**
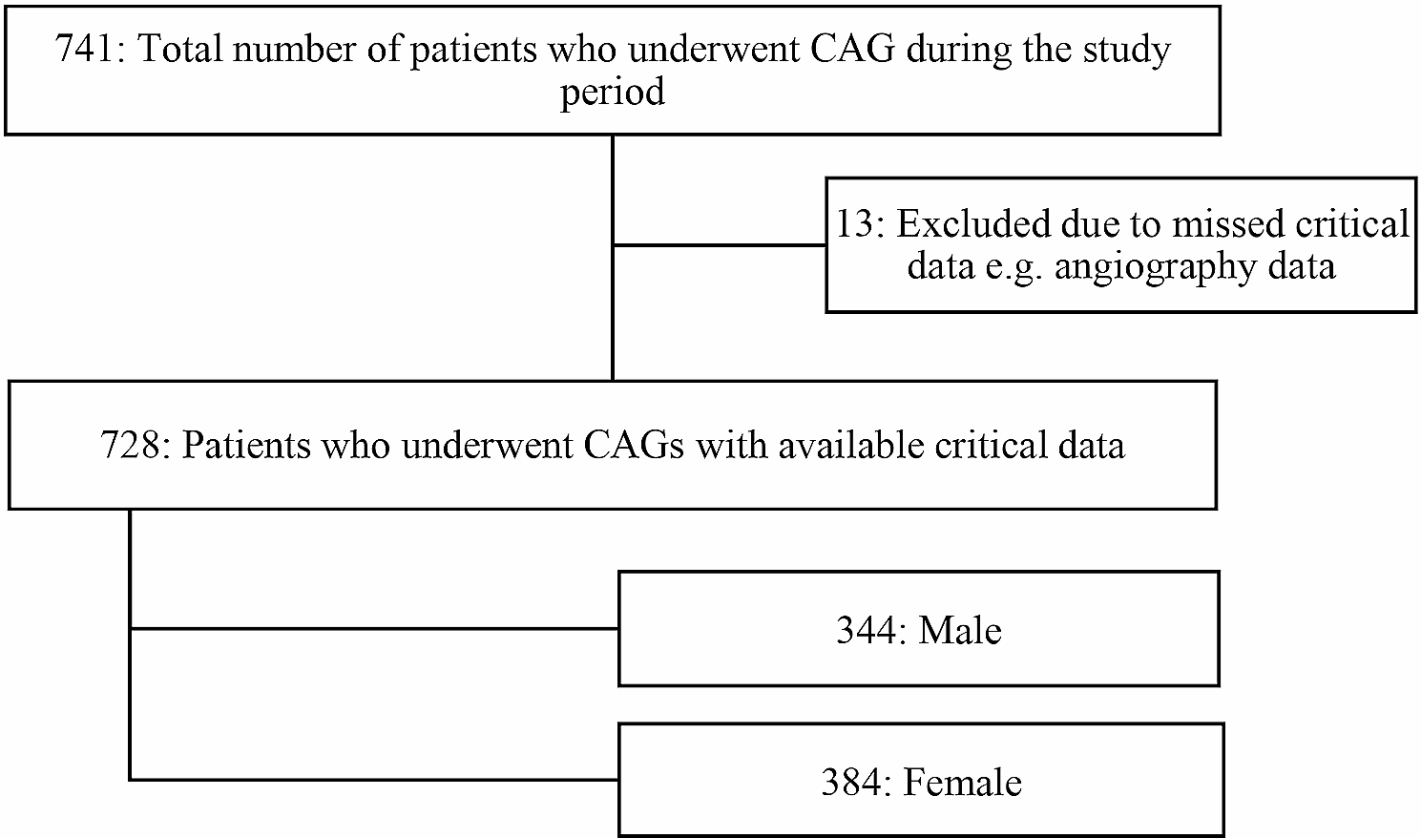
Flow chart for selection of patients from the hospital registry

A total of 741 patients underwent CAG during the period of the study. Thirteen patients were excluded due to incomplete critical data on angiographic records (as depicted in Fig. 1). The remaining 728 patients were included in the final dataset for analysis, of which, 384 (52.23%) were female. The study participants had a sample mean (X̄) age of 59.46 ± 10.83 SD. The patients had a mean body mass index (BMI) of 31.18 kg/m^2^ and nearly a third of patients (28.02%) had an estimated glomerular filtration rate (eGFR) corresponding to chronic kidney disease (CKD) stage 3 and above. The prevalence of hypertension and T2D were estimated at 83.4% and 7.69% respectively (as shown in Table 1).

**Table 1:** Baseline characteristics of study participants who underwent diagnostic coronary angiography

Characteristics		Number, N	Mean (X̄)	SD
**Age**		728	59.46	10.83
**BMI**		656	31.18	17.95
**Serum creatinine**		687	107.65	43.18
**eGFR**		680	72.89	27.08
		***Number, N***	***Percentage (%)***	
**Sex**				
	Male	354	47.77	
	Female	387	52.23	
**Age categories**				
	< 40	31	4.26	
	40–64	463	63.6	
	≥ 65	234	32.14	
**BMI categories**				
Underweight	< 18.5	5	0.76	
Normal	18.5–24.9	142	21.65	
Overweight	25-29.9	212	32.32	
Obese class I	30-34.9	171	26.07	
II	35-39.9	80	12.2	
III	≥ 40	46	7.01	
**Hypertension**				
	Yes	618	83.4	
	No	123	16.6	
**Type 2 diabetes**				
	Yes	57	7.69	
	No	684	92.31	
**CKD stages**				
	1	130	19.2	
	2	356	52.35	
	3 A	128	18.32	
	3B	52	7.65	
	4	12	1.76	
	5	2	0.29	

### Coronary artery angiographic characteristics

The overall prevalence of CAD at any degree of coronary artery obstruction was estimated at 24.86% (34.77% in male, 15.79% in female), while that of obstructive CAD was estimated at 18.27% that is, those with luminal stenosis of at least 50%, the sum of those with moderate and severe luminal stenosis (133 of 728, as shown in Table 2). The CAD prevalence increased with age, with estimates of 16.13% and 35.04% among those below 40 years and above 65 years of age respectively (as shown in Table 2).

Of all patients with obstructive CAD, nearly a half (46.41%) had multiple vessel disease (MVD), of which, 3.87% were found to have triple vessel disease. The majority of patients with affected vessels (76.88%), were found to have significant stenosis. Of these, 20.23% had moderate stenosis (50–70% luminal stenosis) and 56.65% (98 out of 173) had severe stenosis (that is, at least 70% luminal stenosis). Noteworthy, MVD and significant luminal stenosis were higher among male patients compared to female patients. The proportion of patients with MVD was found to be 48.76% in male patients and 41.67% in female patients while that of significant vessel stenosis was found to be 78.45% and 73.68% among male and female patients respectively. About 5.36% of patients were found to have coronary vessel abnormalities which included ecstatic, tortuosity and sluggishly flowing vessels, as shown in Table 2. Being 65 years or older and having comorbidity with T2D were found to be independent predictors of CAD, with a *p* value < 0.001, as highlighted in Table 3.

**Table 2:** Prevalence of obstructive CAD and angiographic characteristics by age and sex among patients who underwent diagnostic coronary angiography

Angiographic characteristics	Sex			Age (years)		
	Male	Female	Total	< 40	50–64	≥ 65
**Vessel outcome**						
Normal vessel	208 (59.77%)	300 (78.95%)	508 (69.78%)	26 (83.87%)	332 (73.61%)	139 (59.40%)
Vessel occlusion	121 (34.77%)	60 (15.79%)	181 (24.86%)	5 (16.13%)	93 (20.62%)	82 (35.04%)
Vessel abnormality	19 (5.46%)	20 (5.26%)	39 (5.36%)	0 (0.0%)	26 (5.76%)	13 (5.56%)
**Number of occluded vessels**						
1	62 (51.24%)	35 (58.33%)	97 (53.59%)	3 (75.00%)	50 (52.63%)	42 (52.50%)
2	52 (42.96%)	25 (41.67%)	77 (42.54%)	1 (25.00%)	40 (42.11%)	36 (45.00%)
3+	7 (5.76%)	0 (0.0%)	7 (3.87%)	0 (0.0%)	5 (5.26%)	2 (2.50%)
**Luminal vessel stenosis**						
Mild: <50%	25 (21.55%)	15 (26.32%)	40 (23.12%)	1 (25.00%)	23 (25.27%)	16 (20.51%)
Moderate: 50–70%	23 (19.83%)	12 (21.05%)	35 (20.23%)	0 (0.0%)	19 (20.88%)	16 (20.51%)
Severe: ≥70	68 (58.62%)	30 (52.63%)	98 (56.65%)	3 (75.00%)	49 (53.85%)	46 (58.97%)

**Table 3:** Logistical regression modelling for independent variables for developing obstructive coronary artery disease

Independent Variables	Odds Ratio (OR)	Standard Error (SE)	Z	P >│z│	95% Confidence Interval
Age ≥ 65 years	1.86	0.35	3.32	0.001	1.29–2.67
Male gender	2.68	0.50	5.25	0.000	1.85–3.87
Overweight	0.40	0.07	-5.02	0.000	0.28–0.57
Type 2 diabetes	2.62	0.78	3.24	0.001	1.85–4.70
Hypertension	1.16	0.26	0.68	0.497	0.75–1.81

**Fig. 2:**
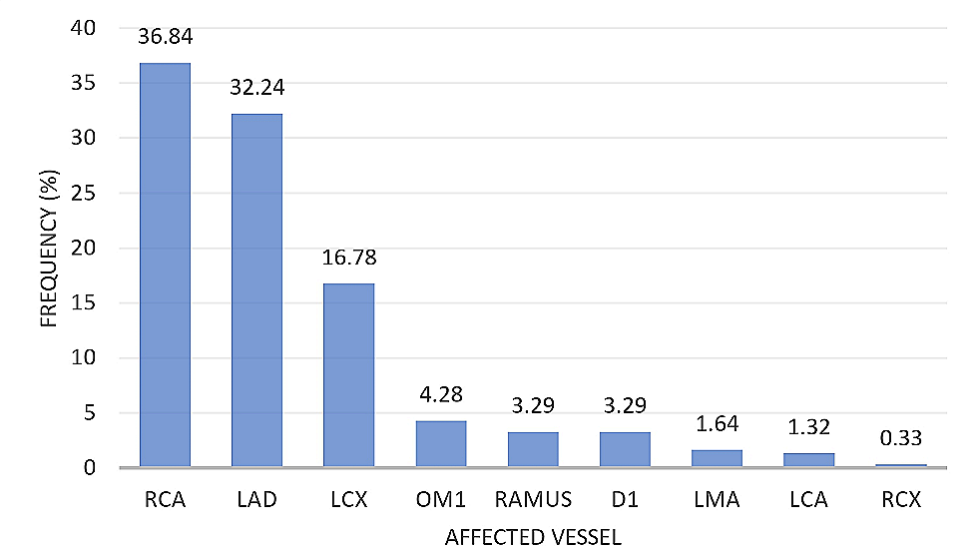
Frequency distribution of the anatomical vessel involvement in patients with ‘any vessel disease’. RCA – right coronary artery, LAD – left anterior descending artery, LCx – left circumflex artery, OM1 – oblique main, D1 – diagonal branch, LMA – left main artery, LCA – left coronary artery, and RCx – right circumflex artery

**Fig. 3:**
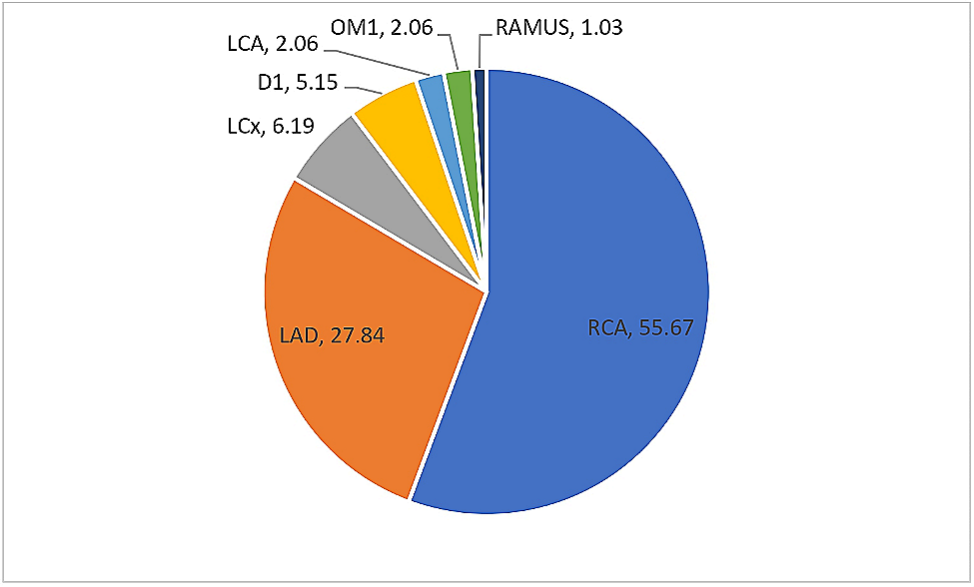
Percentage frequency distribution of the anatomical vessel involvement in patients with ‘single vessel disease’

Notwithstanding the number of vessels affected, of the affected coronary vessels, RCA had the highest frequency of 36.84%, followed by left anterior descending (LAD) artery and left circumflex (LCx) artery which had a frequency of 32.24% and 16.78% respectively, as highlighted in Fig. 2. Moreover, when only single vessel disease is considered, RCA still remained the most commonly affected vessel, being affected in about 56% of all patients, followed by LAD artery which was affected in 28% of cases, as depicted in Fig. 3.

## Discussion

The prevalence of obstructive CAD of any degree of coronary artery luminal stenosis was estimated at 24.86% among patients undergoing diagnostic CAG in a tertiary care facility in Tanzania. This is comparable to a similar study conducted in Cameroon, which reported the prevalence of 28.8% among patients who underwent diagnostic CAG [17]. Similarly, our findings are comparable to a study conducted in Iran which revealed the prevalence of 19.1% based on a combination of clinical evaluation and CAG findings [18]. Noteworthy, a similar study by Khanbhai, KS et al. conducted at Jakaya Kikwete Cardiac Institute (JKCI); another centre in a more urbanized population in Tanzania, reported the prevalence of 45.5% [19]. The reasons for the observed discrepancy are not entirely clear, however, two factors could be speculated as the possible explanations. Firstly, it is noteworthy that CAG services are expensive and largely unaffordable to most patients in Tanzania; only affordable to those with high insurance premium coverage. Thus, patients with suspected CAD without high premium insurance coverage are less likely to undergo CAG and subsequent coronary intervention, which implies that the nature of patients who undergo cardiac catheterization are often not the true representative of the population. Moreover, even those patients who are insured, for patients in whom MVD is diagnosed, most of the insurance companies would cover the cost for only one stent at a sitting when percutaneous coronary intervention (PCI) is indicated. Secondly, the two cities – Dar es Salaam and Dodoma – where the two “Cath Lab” centres are located might differ in the level of urbanization and associated risk factors for ASCVD [19, 20]. JKCI, being a national cardiovascular centre, tends to receive referral patients with advanced CAD from across hospitals in the country for diagnostic CAG and coronary intervention. Similarly, the increasing medical tourism from neighbouring countries of patients with established, and possibly with severe CAD could potentially over-represent the prevalence of obstructive CAD at JKCI.

The prevalence of CAD was noted to have male gender preponderance and increasing with age consistent with studies conducted elsewhere, including analysis of global data by Khan MA et al. [5], and in a review by Shehu, MN et al. [11]. Age is a well-established independent risk factor for developing NCDs, CAD being not an exceptional. The notably higher prevalence of CAD among male compared to female is multifaceted. This could partly be attributed to the presence of competing risk factors for CAD which are associated with the male gender, particularly smoking and alcohol consumption which are both known to be higher among men in SSA. For instance, a review by Magitta, NF indicates smoking and tobacco use to be primarily a behavioral risk among men in the majority of countries in SSA; with the prevalence of 17.5% and 2.2% among men and women respectively, a pattern which is similar across countries worldwide [21].

The majority of patients with CAD had hypertension and a few of them had T2D, which are well known independent risk of developing CAD. However, hypertension showed a trend towards CAD risk, though it was not shown to be statistically significant contrary to other previous studies [4, 11]. The reason for this observation remain elusive, however, a small sample size could have an impact on the power of the study. T2D is a well-established risk factor for developing CAD, as affirmed by this study in parallel to other previously reported studies. T2D could operate through multiple mechanisms which include increased oxidative stress, endothelial injury, lipid peroxidation and ultimately atherosclerosis. Moreover, the co-existence of hypertension in the context of metabolic syndrome frequently exert synergistic effects to promote the development of CAD [11, 17].

In this study over three-quarters of patients with angina chest pain were found to have angiographically normal coronary arteries. The majority of these patients could have non-ischemic chest pain arising from other causes. However, a minority of patients with typical ischemic chest pain could potentially have non-obstructive CAD; what is termed as ischemia with non-obstructive coronary artery (INOCA), *alias* angina with non-obstructive CAD (ANOCA). It is generally well established that up to 70% of patients with CAD may not have coronary artery obstruction, though in patients undergoing diagnostic CAG, the absence of obstruction may either rule out CAD or potentially rule in a possible diagnosis of INOCA [15]. The latter is relatively common among women (up to 50–70%) compared to men (30–50%) [15]. In our study, women contributed about 60% of those without coronary obstruction, indicating a possible diagnosis of INOCA in some of these patients.Thus,  our findings are in parallel to a study conducted in Poland among young patients aged below 40 years with a diagnosis of CAD which revealed angiographically normal coronary arteries in 37.2% of those with CAD and 16.9% of those with ACS [22]. The mechanisms underlying INOCA/ANOCA/MINOCA are diverse but available evidence suggests several endotypes that include coronary microvascular dysfunction, augmented vasoreactivity or spasm, non-obstructive atherosclerosis, pre-heart failure with preserved ejection fraction, and hypercoagulability which are either acting singly or in combination [23, 24]. Moreover, there is a body of evidence on racial variability on the severity of CAD, such that black patients with coronary insufficiency have been shown to be more likely to have ANOCA/INOCA compared to other races [25, 26]. This could therefore partly explain the reason for the observed low prevalence of obstructive CAD in our study compared to the findings in other reports.

Noteworthy, about 5.36% of patients were found to have coronary vessel abnormalities which include ecstatic, tortious and sluggishly flowing vessels, in agreement with previous reports [27]. These aberrant vessels could be associated with inadequate blood supply to meet the myocardial demand resulting in ischemic pain. The mechanisms for the vascular aberrations are not well understood. However, developmental anomalies during embryological morphogenesis due to hitherto unknown causes could be the underlying insults [27]. Recently, overexpression of vascular endothelial growth factor – beta (VEGF-β) in mice has been shown to promote the proliferation of endothelial cells and coronary vessels in the embryological heart development [28]. This signaling pathway is currently being explored as a potential therapeutic target for reperfusion therapy. We further postulate that in SSA, where there is widespread, repeated malaria infection particularly during childhood, the associated oxidative stress and pro-inflammatory cytokines could trigger subtle changes through activation of nuclear factor kappa – beta (NF-κβ) pathway in the vascular system including coronary vessels that could lead to their developmental aberration [29].

Over a half (53.59%) of patients had single vessel disease, slightly lower compared to 61.9% reported in a study conducted in Poland [22]. A substantial proportion of patients (46.41%) displayed MVD, of which 3.87% were found to have triple vessel disease. Likewise, this population of patients further demonstrated a severe degree of vessel stenosis which revealed about 76.88% of patients to have coronary artery luminal stenosis of at least 50% while those who had over 70% degree of stenosis alone accounted for 56.65%. The reasons for this observation are not readily discernable. However, we speculate that patients in this study might have had symptoms for longer time without seeking medical attention or inadvertently could have sustained unrecognized or missed myocardial infarction without being evaluated for CAD. Similarly, this observation could further be attributable to late diagnosis partly due to lack of access to CAG services in Tanzania. It should be recalled that CAG services are relatively new in the majority of countries in SSA, with only two centres in Tanzania – with our centre being operational for less than three years [16]. It is well settled that the presence of multiple risk factors in an individual patient contributes substantially to the vessel pathology which orchestrate the development of ASCVD.

The most common affected vessel in this study was an RCA – which was involved in  36.84% and at 55.67% when single vessel disease was considered. This finding is in agreement with other studies conducted elsewhere. For instance, a study conducted in Egypt among patients with ST segment elevation myocardial infarction (STEMI) indicated RCA to be most commonly affected vessel in 34.6% of patients [30]. However, this finding is contrary to other studies which revealed LAD as the predominant affected vessel. For instance, studies conducted in Cameroon, China and Poland reported LAD artery as the most commonly affected vessel, accounting for 42.28%, 45.5% and 61.6% respectively [17, 22, 31]. The type of the affected vessel could have a clinical significance as the occlusion of RCA could potentially affect the blood supply to the sinoatrial node and the conducting system of the heart resulting in rhythm disturbances and conduction defects. The reason for the observed differences in the predilection of CAD affected vessel remains unknown. However, the existing body of evidence indicates subtle anatomical variation in the coronary circulation among human races [32]. Though, in his seminal work, R. Singer in 1959 did not find any significant racial differences in the major coronary branches [33], skeptics exist on the human variability in the coronary artery luminal diameters. However, it is not known whether any potential anatomical variation could entirely explain the observed inter-racial coronary atherosclerotic predilection and disease severity.

There are two major limitations for this study. Firstly, the study being retrospective it was susceptible to incompleteness or loss of data. This happened because specific variables were not initially considered important and were therefore, not collected or due to limited hospital archiving capacity, some variables were not available particularly when hardcopies were used for initial data collection, though the hospital largely uses electronic data capture and storage platforms. Secondly, CAG services are expensive and not readily accessible or affordable to the majority of patients without adequate insurance premiums. Thus, the data largely consist of patients who are either ensured or those from high socio-economic status, hence without adequate representativeness of the population and with limited generalizability to the wider population.

## Conclusion

The prevalence of CAD is substantial among patients undergoing diagnostic CAG. The male gender and age are important non-modifiable risk factors for developing CAD. The current study shows that patients often display MVD and frequently have severe coronary artery luminal stenosis. A more detailed study on the assessment of risk factor profile and CAG correlates is needed to provide more insightful information for designing a tailored public health intervention for preventive cardiology in limited resource settings in SSA.

## Data Availability

The datasets generated and/or analyzed during the current study are not publicly available due to local ethical restriction related to patients’ confidentiality but are available from the corresponding author on reasonable request.
